# Supplementary material for: First-Generation EGFR-TKI Plus Chemotherapy Versus EGFR-TKI Alone as First-Line Treatment in Advanced NSCLC With EGFR Activating Mutation: A Systematic Review and Meta-Analysis of Randomized Controlled Trials
Source: Front Oncol. 2021 Apr 13;11:598265. doi: 10.3389/fonc.2021.598265 (PMC8076535; doi:10.3389/fonc.2021.598265)
Supplement: Supplementary file 2 [file Table_2.docx]

**S1 Fig. Risk of bias summary or review of judgements about each risk of bias item for each included study (A) and presented as percentages across all included studies (B).**


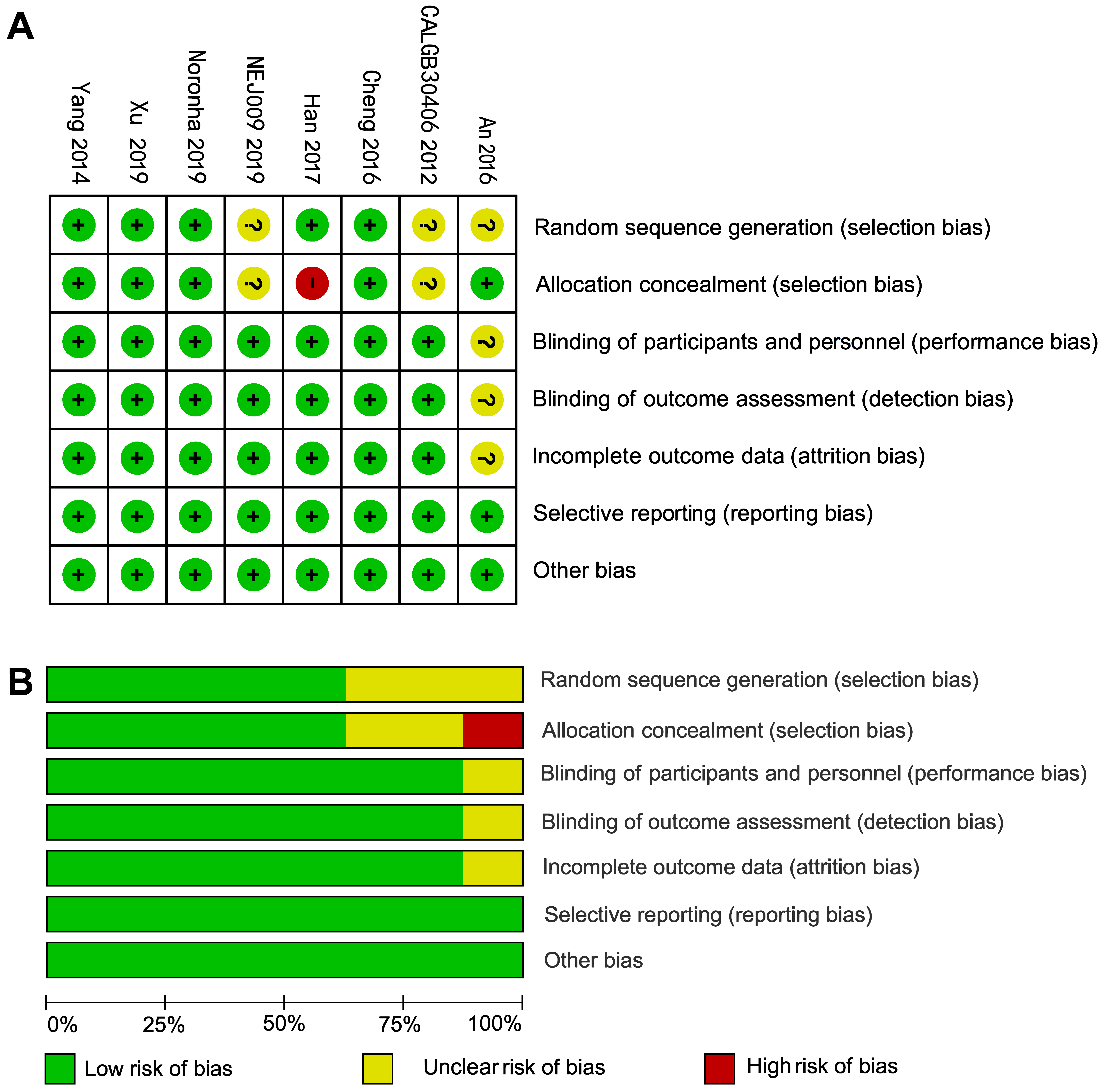


**S2 Fig. Funnel plot of publication bias of progress-free survival (A) and overall survival (B).**


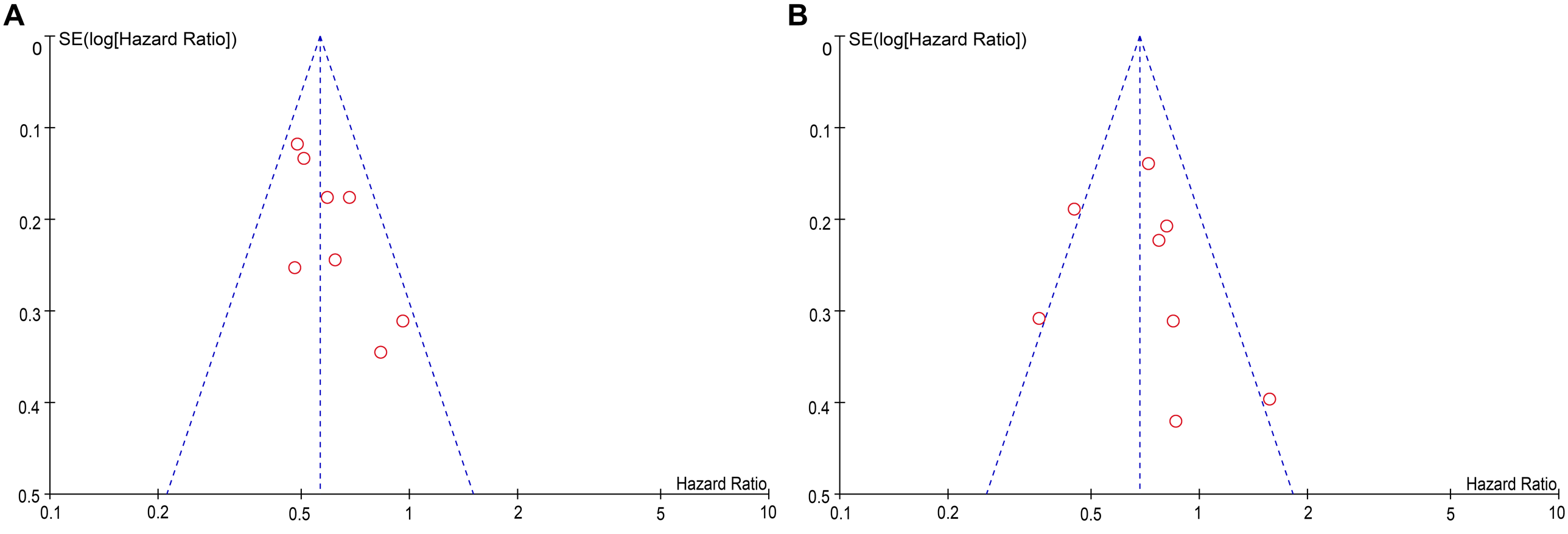


**Supplementary selection of study:** 13 potential studies were excluded because of retrospective studies [1-4], unselected EGFR mutation [5-8], single arms [9-11], small sample size [12], and insufficient survival data [13].

1. Liu S, He Y, Jiang T, Ren S, Zhou F, Zhao C, et al. EGFR-TKIs plus chemotherapy demonstrated superior efficacy than EGFR-TKIs alone as first-line setting in advanced NSCLC patients with EGFR mutation and BIM deletion polymorphism. Lung cancer (Amsterdam, Netherlands). 2018;120:82-7. Epub 2018/05/12. doi: 10.1016/j.lungcan.2018.04.004. PubMed PMID: 29748021.

2. Wang L, Li Y, Li L, Wu Z, Yang D, Ma H, et al. The effect of icotinib combined with chemotherapy in untreated non-small-cell lung cancer that harbored EGFR-sensitive mutations in a real-life setting: a retrospective analysis. OncoTargets and therapy. 2018;11:2345-53. Epub 2018/05/08. doi: 10.2147/ott.s157755. PubMed PMID: 29731642; PubMed Central PMCID: PMCPMC5927338.

3. Wen M, Xia J, Sun Y, Wang X, Fu X, Zhang Y, et al. Combination of EGFR-TKIs with chemotherapy versus chemotherapy or EGFR-TKIs alone in advanced NSCLC patients with EGFR mutation. Biologics : targets & therapy. 2018;12:183-90. Epub 2018/12/18. doi: 10.2147/btt.s169305. PubMed PMID: 30555222; PubMed Central PMCID: PMCPMC6280904.

4. Yan X, Wang H, Li P, Zhang G, Zhang M, Yang J, et al. Efficacy of first-line treatment with epidermal growth factor receptor-tyrosine kinase inhibitor (EGFR-TKI) alone or in combination with chemotherapy for advanced non-small cell lung cancer (NSCLC) with low-abundance mutation. Lung cancer (Amsterdam, Netherlands). 2019;128:6-12. Epub 2019/01/16. doi: 10.1016/j.lungcan.2018.12.007. PubMed PMID: 30642455.

5. Gatzemeier U, Pluzanska A, Szczesna A, Kaukel E, Roubec J, De Rosa F, et al. Phase III study of erlotinib in combination with cisplatin and gemcitabine in advanced non-small-cell lung cancer: the Tarceva Lung Cancer Investigation Trial. Journal of clinical oncology : official journal of the American Society of Clinical Oncology. 2007;25(12):1545-52. doi: 10.1200/JCO.2005.05.1474. PubMed PMID: 17442998.

6. Giaccone G, Herbst RS, Manegold C, Scagliotti G, Rosell R, Miller V, et al. Gefitinib in combination with gemcitabine and cisplatin in advanced non-small-cell lung cancer: a phase III trial--INTACT 1. Journal of clinical oncology : official journal of the American Society of Clinical Oncology. 2004;22(5):777-84. doi: 10.1200/JCO.2004.08.001. PubMed PMID: 14990632.

7. Herbst RS, Giaccone G, Schiller JH, Natale RB, Miller V, Manegold C, et al. Gefitinib in combination with paclitaxel and carboplatin in advanced non-small-cell lung cancer: a phase III trial--INTACT 2. Journal of clinical oncology : official journal of the American Society of Clinical Oncology. 2004;22(5):785-94. doi: 10.1200/JCO.2004.07.215. PubMed PMID: 14990633.

8. Herbst RS, Prager D, Hermann R, Fehrenbacher L, Johnson BE, Sandler A, et al. TRIBUTE: a phase III trial of erlotinib hydrochloride (OSI-774) combined with carboplatin and paclitaxel chemotherapy in advanced non-small-cell lung cancer. Journal of clinical oncology : official journal of the American Society of Clinical Oncology. 2005;23(25):5892-9. Epub 07/25. doi: 10.1200/JCO.2005.02.840. PubMed PMID: 16043829.

9. Kanda S, Horinouchi H, Fujiwara Y, Nokihara H, Yamamoto N, Sekine I, et al. Cytotoxic chemotherapy may overcome the development of acquired resistance to epidermal growth factor receptor tyrosine kinase inhibitors (EGFR-TKIs) therapy. Lung cancer (Amsterdam, Netherlands). 2015;89(3):287-93. Epub 07/02. doi: 10.1016/j.lungcan.2015.06.016. PubMed PMID: 26169499.

10. Tamiya A, Tamiya M, Shiroyama T, Saijo N, Nakatani T, Minomo S, et al. Phase II trial of carboplatin, S-1, and gefitinib as first-line triplet chemotherapy for advanced non-small cell lung cancer patients with activating epidermal growth factor receptor mutations. Med Oncol. 2015;32(3):40-. Epub 01/25. doi: 10.1007/s12032-014-0474-x. PubMed PMID: 25616723.

11. Yoshimura N, Kudoh S, Mitsuoka S, Yoshimoto N, Oka T, Nakai T, et al. Phase II study of a combination regimen of gefitinib and pemetrexed as first-line treatment in patients with advanced non-small cell lung cancer harboring a sensitive EGFR mutation. Lung cancer (Amsterdam, Netherlands). 2015;90(1):65-70. Epub 06/15. doi: 10.1016/j.lungcan.2015.06.002. PubMed PMID: 26238424.

12. Hirsch FR, Kabbinavar F, Eisen T, Martins R, Schnell FM, Dziadziuszko R, et al. A randomized, phase II, biomarker-selected study comparing erlotinib to erlotinib intercalated with chemotherapy in first-line therapy for advanced non-small-cell lung cancer. Journal of clinical oncology : official journal of the American Society of Clinical Oncology. 2011;29(26):3567-73. Epub 08/08. doi: 10.1200/JCO.2010.34.4929. PubMed PMID: 21825259.

13. Yang H, Deng Q, Qiu Y, Huang J, Guan Y, Wang F, et al. Erlotinib intercalating pemetrexed/cisplatin versus erlotinib alone in Chinese patients with brain metastases from lung adenocarcinoma: a prospective, non-randomised, concurrent controlled trial (NCT01578668). ESMO Open. 2017;2(Suppl 1):e000112-e. doi: 10.1136/esmoopen-2016-000112. PubMed PMID: 29147576.
